# On-chip flow cytometer using integrated photonics for the detection of human leukocytes

**DOI:** 10.1038/s41598-024-60708-0

**Published:** 2024-05-20

**Authors:** Stijn Jooken, Kirill Zinoviev, Günay Yurtsever, Anabel De Proft, Koen de Wijs, Zeinab Jafari, Ana Lebanov, Gaudhaman Jeevanandam, Mateusz Kotyrba, Erwin Gorjup, Jelle Fondu, Liesbet Lagae, Sarah Libbrecht, Pol Van Dorpe, Niels Verellen

**Affiliations:** 1 imec, Kapeldreef 75, 3001 Leuven, Belgium; 2 Sarcura GmbH, Plöcking 2, Klosterneuburg, Austria

**Keywords:** Biophotonics, Optofluidics, Integrated optics, Flow cytometry

## Abstract

Differentiation between leukocyte subtypes like monocytes and lymphocytes is essential for cell therapy and research applications. To guarantee the cost-effective delivery of functional cells in cell therapies, billions of cells must be processed in a limited time. Yet, the sorting rates of commercial cell sorters are not high enough to reach the required yield. Process parallelization by using multiple instruments increases variability and production cost. A compact solution with higher throughput can be provided by multichannel flow cytometers combining fluidics and optics on-chip. In this work, we present a micro-flow cytometer with monolithically integrated photonics and fluidics and demonstrate that both the illumination of cells, as well as the collection of scattered light, can be realized using photonic integrated circuits. Our device is the first with sufficient resolution for the discrimination of lymphocytes and monocytes. Innovations in microfabrication have enabled complete integration of miniaturized photonic components and fluidics in a CMOS-compatible wafer stack. In combination with external optics, the device is ready for the collection of fluorescence using the on-chip excitation.

## Introduction

With the successful implementation of cell therapy in clinical practice, there is a need to increase the throughput and automation of cell analysis and isolation techniques^1^. To guarantee the delivery of functional cells, cell therapies rely on the controlled processing of billions of cells by trained specialists in a limited timespan to ensure qualitative, cost-effective, and timely delivery to the patient^2,3^. Currently, magnetic bead isolation is the most commonly used FDA-approved therapy due to constraints regarding good manufacturing practices (GMP) and the requirement for high throughput^4–6^. Yet, this technique is limited to single parametric isolation and lacks in-line quality control of the input sample. Alternatively, fluorescence-activated cell sorting (FACS), a flow cytometry technique, is available. Typically, GMP-compliant FACS devices do not reach the high throughputs achieved in state-of-the-art FACS devices ^7^. Nevertheless, the use of FACS for cell isolation does give the advantage of multiparametric process control and in-line quality control based on visible light scattering as well as one or more fluorescence profiles. In FACS, immunofluorescence, which is the detection of fluorescence emission of antibody-dye complexes bound to cell-type specific surface receptors, enables unique cell identification and subsequent sorting. State-of-the-art devices are equipped with several lasers for the excitation of multiple markers, either coinciding or spatially separated along the fluidic stream, and can detect emission from a few dozen fluorophores per cell^8^. Light scattering, on the other hand, inherent to flow cytometry, provides an additional, label-free analysis modality for mapping a sample’s composition. The intensities of the forward- and side-scattered light carry information on a cell’s size and granularity, respectively, and allow identification of target cell populations, such as lymphocytes or monocytes, and the presence of dead or unwanted populations without additional effort^9^.

The maximum detection rate of flow cytometers typically depends on the sample flow speed, the maximum sample concentration, the power of the light source, and the signal-to-noise ratio (SNR) of the readout module. Additionally, factors related to automation and assay design contribute to the overall performance. Nonetheless, when applied for cell sorting, it is the sorting module that primarily dictates the throughput. Once the maximum sorting rate is achieved, the implementation of parallelization across multiple channels becomes essentially the most promising approach to further enhance throughput^1^. In this context, the adoption of on-chip flow cytometry with monolithically integrated optical components presents an attractive solution. Apart from parallelization, it offers various other advantages, including a compact, robust, and disposable design and alignment-free operation of the interrogation point.

During the last two decades, several on-chip flow cytometers have been fabricated by soft lithography in polymers with integrated refractive optics for illumination and grooves for the insertion of optical fibers for collection^10–14^. The main drawback of these devices lies in the manual assembly, making them expensive for mass production. Integrated devices, with both microfluidics and photonics fully integrated on-chip, have been demonstrated as well, but their operation has been limited to the discrimination of microbeads or cell counting^15–18^. Nowadays, technology platforms for the fabrication of integrated photonics with high refractive index materials are becoming standard. These materials, such as silicon and silicon nitride, are optimized for compact light routing and low propagation loss. Silicon nitride furthermore allows operation in the visible range with low auto-fluorescence, while showing promise to incorporate light sources, detectors, and other active devices in the near future^19,20^. Photonics on-chip has also been demonstrated to allow the illumination of particles with tailored beam shapes^21–23^, but the feasibility of collecting scattered light using integrated waveguides has not yet been studied. In principle, integrated waveguides cannot compete with bulk optics (high NA lenses) or even multimode fibers in terms of collection efficiency due to the small waveguide cross-section. The advantage of using waveguides lies mainly in (1) the alignment-free routing of light collected at an interrogation point to another point on-chip, where it can be processed, either by external optical components and detectors or by integrated detectors, and (2) the cost-effective batch-fabrication. The co-integration of sources, detectors, and front-end electronics onto disposable flow cytometer chips would increase the price of the devices. However, the cost-to-performance ratio should be estimated, considering how many devices can be allocated on-chip, and what benefits in terms of packaging, calibration, and maintenance it would bring.

In this work, we demonstrate a fully functional device for on-chip flow cytometry with monolithically integrated waveguide optics and fluidics. The optical device is capable of detecting the side- and forward-scattered light of white blood cells, with sufficient resolution for the discrimination of monocytes and lymphocyte populations in a peripheral blood mononuclear cell (PBMC) sample. Moreover, the immunofluorescent detection of these populations was possible using the on-chip excitation and external optics for collection.

The chip was fabricated using microelectronic CMOS-compatible technologies and was designed for high-volume production. Specifically, recent advances in micro-electro-mechanical systems and microelectronics technologies permitted the chip to be constructed by bonding three wafers, of which two contain integrated photonics. The on-chip integration of fluidic and photonic structures as such allows for GMP compliance, as the resulting closed-loop system reduces system variability with in-line sample monitoring. In addition, it enables high throughput by parallelization. Moreover, the optical and fluidic configuration is suitable for future integration with our previously developed on-chip bubble jet sorter ^24^.

## Results and discussion

This section starts by introducing the on-chip flow cytometer, discussing details of the microfluidic components, the illumination-, and collection photonics. Next, data on the detection of polystyrene beads as a calibration standard is presented. Subsequently, to evaluate the performance for a cell therapy manufacturing workflow, data from PBMCs is discussed. For a reliable, high-quality read-out, comparable to conventional flow cytometry, the device must be capable of discriminating the two major PBMC subpopulations, monocytes and lymphocytes. To determine the specific scatter signals collected for either cell subtype, first, magnetic bead isolated monocytes and lymphocytes were used. The discrimination of isolated lymphocytes and monocytes is then validated on a full PBMC sample. Next, the effect of design variations in the collection optics on the collected signals and the device performance is discussed. Finally, immunofluorescent detection is demonstrated.

### The on-chip flow cytometer

**Figure 1:**
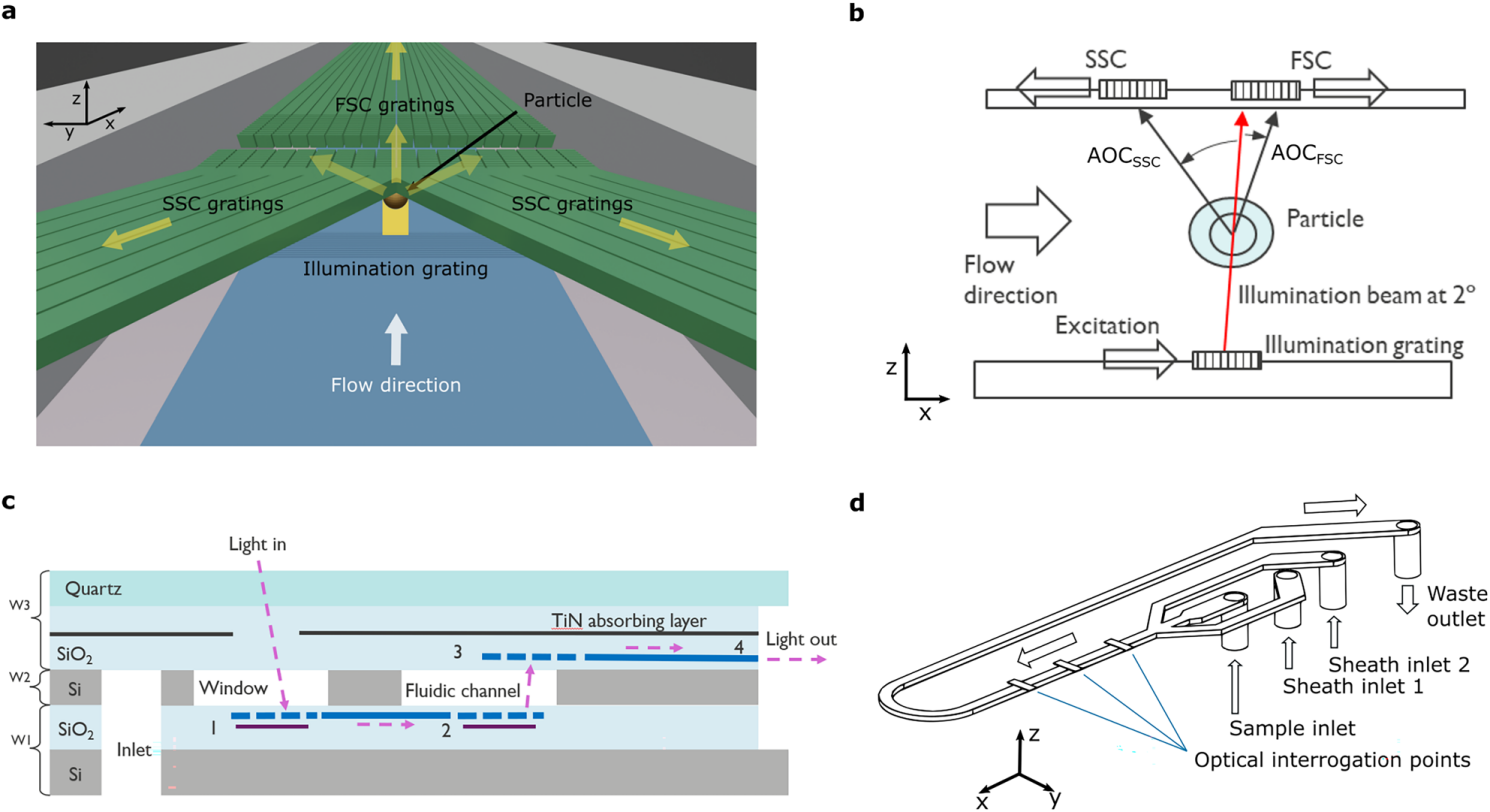
Schematic illustrations of the on-chip flow cytometer. (**a**) The distribution of the optical components at the interrogation point. (**b**) Schematic illustration of the layout of the optical detection. (**c**) Cross-section of the layer stack, indicating the main components of the on-chip flow cytometer: 1—in-coupling grating, 2—illumination grating, 3—collection gratings, 4—edge-coupler on quartz, W1—bottom silicon wafer, W2—middle silicon wafer, W3—top quartz wafer. The drawing is not to scale. (**d**) A schematic drawing of the fluidic components. Several optical interrogation points are located along the microfluidic channel. The sheath flows allowed fluidic side focusing of the sample flow and were monitored using flow meters and pressure sensors.

At the interrogation point, highlighted in Fig. 1**a-b**, particles pass through the excitation light, which is directed upwards by the illumination grating. The light that is scattered by the particle is then locally collected by the collection gratings placed in the top quartz layer. The chip stack is shown schematically in Fig. 1**c**. It contains fluidic inlets and illumination photonics in the bottom silicon wafer, microfluidic channels in the middle silicon wafer, and collection photonics in the top quartz wafer. In this report, the gratings for the collection of scattering in the forward direction are called FSC gratings, and those for side scattering collection are called SSC gratings. The transparent quartz top wafer enabled optical access for visual inspection, characterization of individual structures, measuring illumination power at the interrogation point, and fluorescence collection.

### Fluidics

The middle wafer hosts the microfluidic channels, accessible through in- and outlets etched in the bottom silicon wafer and sealed from the top by the quartz. The fluidic channels are shown schematically in Fig. 1**d**. The microfluidics consist of 2 side channels for the sheath flows and 1 for the sample flow, which are joined at a Y-junction to enable 2D fluidic focusing of the sample flow. The focused sample then passes through a channel with a cross-section of 30 × 100 µm^2^ (height × width) and a length of 15 mm. Several optical interrogation points are located along the channel which allows to evaluate different device variations within a single fluidic channel. Finally, the sample exits the chip through the waste outlet. We define the coordinate axes as shown in Fig. 1**a**: in flow direction (X), in-plane across the flow (Y), and vertical across the flow (Z).

### Illumination photonics

**Figure 2:**
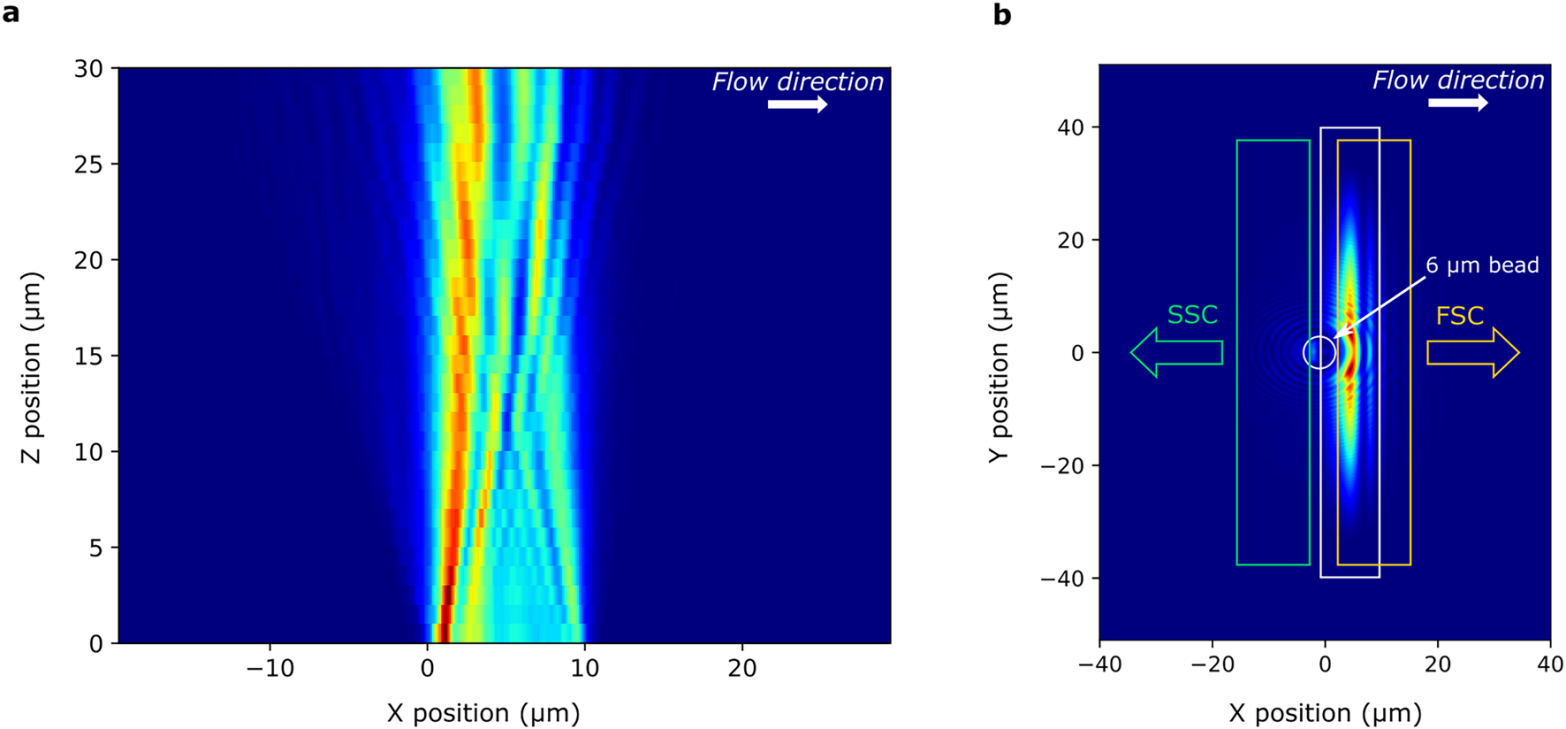
The illumination beam profile. (**a**) Experimentally measured 2D intensity profile of the illumination beam in the center of the channel parallel to the flow (XZ-plane). (**b**) The layout of collection gratings is sketched on top of a simulated distribution of the intensity distribution of the electric field component |E_y_|^2^ (the component perpendicular to the flow and parallel to the plane of the chip) at the plane of the gratings on quartz calculated using a 3D FDTD model built for a 6 µm polystyrene bead entering the illumination beam.

The illumination photonics have been designed based on a SiN platform for red light which offers low losses and low auto-fluorescence. The excitation light with a wavelength of 638 nm is coupled into the chip using a diffraction grating coupler and then delivered via a waveguide to the illumination grating. The latter is a linear diffraction grating with dimensions of 10 × 70 µm^2^ (along and across the flow), which shoots the light up through the fluidic channel at an angle of 2° with respect to the normal. Both the in-coupling and illumination gratings have an aluminum reflector underneath to increase the coupling efficiencies. The illumination beam has a quasi-Gaussian intensity profile in the ‘across the flow’-plane (YZ-plane) with a width of 50 µm (measured at 1/e^2^). In the direction parallel to the flow (XZ-plane), the beam has an irregular quasi-exponential profile with a main lobe and a plurality of side lobes, which can be seen in the experimentally measured profile presented in Fig. 2**a**.

### Collection photonics

Both the FSC and SSC gratings are fully etched linear diffraction gratings, coupling the collected light to multimode (MM) waveguides. The waveguides transmit the collected light to edge couplers, where it is picked up with fiber optics. The MM waveguides were designed to have a height of 180 nm and a width of 2.5 µm to balance optical throughput and device size, taking into account bending radius limitations and practical constraints. The positions of the collection gratings in the top quartz layer were defined by geometrical optics. Figure 1**b** schematically illustrates the angle of collection (AOC), that is the angle with respect to the illumination beam, for both the FSC and SSC gratings. Two different FSC geometries were fabricated. In the first, light is collected at an AOC of 5°, while in the second, light is collected at an AOC of 0° (corresponding to 2° with respect to the normal). The latter corresponds to detection in axial light-loss (ALL) mode and not scattering. This method has been reported to be applied as an alternative to forward scatter in commercial devices ^25^. ALL relies on the measurement of the light remaining in the illumination beam during its interaction with a cell^26^, rather than a measurement of the forward scattered light by the cell. Despite a lower sensitivity, it was demonstrated that this optical parameter provides better resolution of human leukocytes than the combination of other light scattering measurements^25^. In this work, collection gratings for ALL will still be called ‘FSC gratings’ for consistency with conventional terminology. For SSC, on the other hand, collection gratings were designed for an AOC of 40°, 50° and 70°. In the results section, only data collected at an interrogation point of a so-called “nominal configuration design” with FSC collected at 0° (ALL) and SSC collected at 50° is presented. Experimental results on the other configurations are shown in the supplementary information (SI).

Since the grating-particle distance is comparable to the size of the particle, the collection gratings act as near-field probes of the scattered light. Figure 2**b** shows an overlay of the gratings, represented by rectangles, with the intensity distribution of the electric field component |E_y_|^2^ (equivalent to the power distribution) obtained for a 6 µm polystyrene bead passing through the illumination beam using full-field 3D Finite Difference Time Domain (FDTD) modeling. The resulting field contains submicron features with particle position- and time-dependent intensities and polarizations. For a particle going through the center of the channel, the scattering pattern is symmetrical and as such, for efficient collection, the gratings could be symmetrically distributed along the perimeter of a circle. However, to minimize the dependence of the signal on the particle’s Y-position (across the flow), the SSC grating array was arranged in a line across the microfluidic channel. Moreover, due to the side lobes of the illumination beam, a small portion of the illumination light is directed toward the SSC gratings. Although it is low-power light, it creates a background signal comparable to the SSC signal, which affects the SNR. Typical numbers for the background signals can be found in the SI. In total, 18 FSC gratings and 18 SSC gratings were positioned at the interrogation point.

### Detection of polystyrene beads

**Figure 3:**
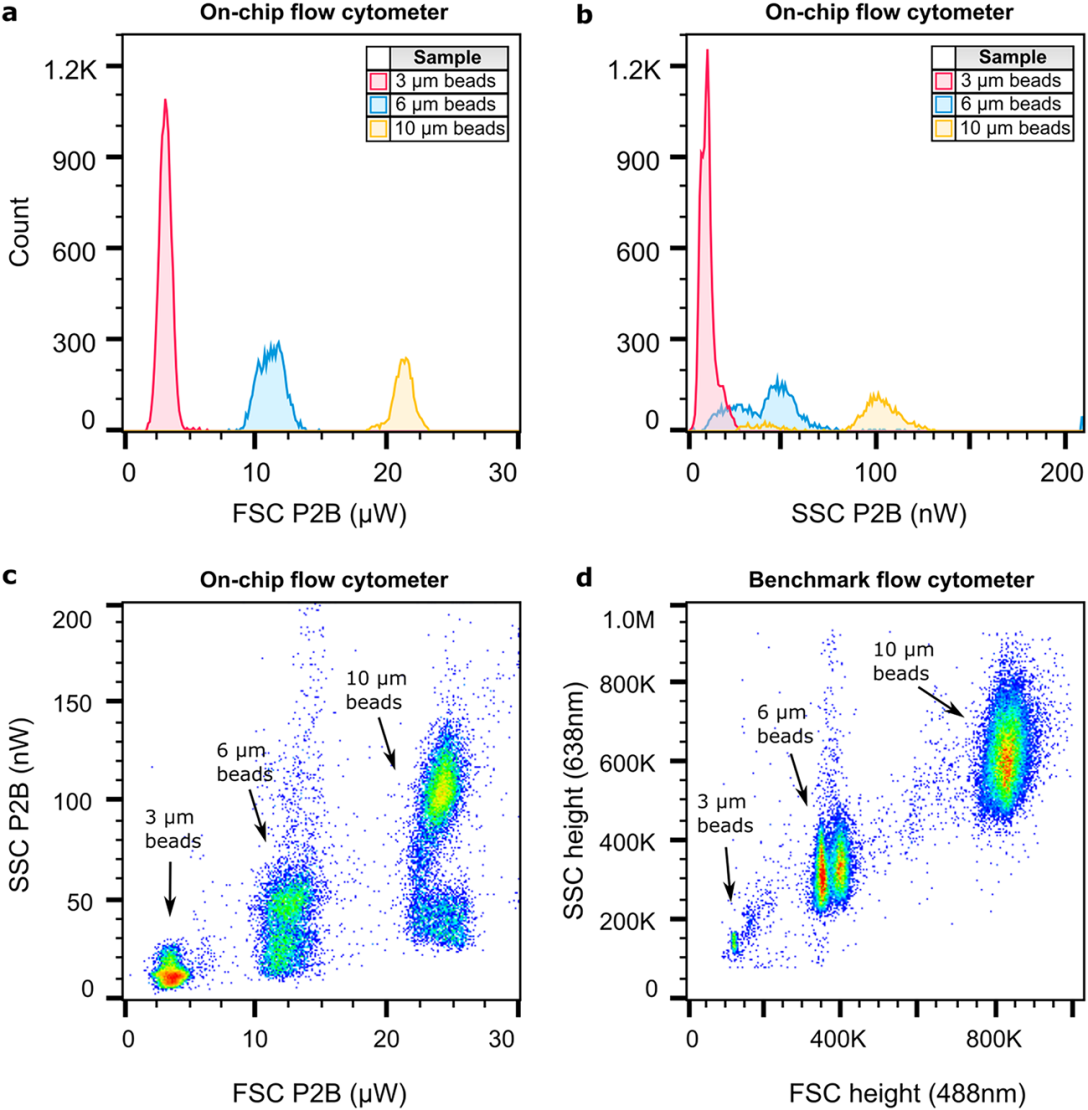
Counting and discrimination of polystyrene bead populations on-chip and on a benchmark flow cytometer. Histograms of the peak-to-baseline (P2B) amplitude of (**a**) FSC and (**b**) SSC signals of 3 µm, 6 µm, and 10 µm beads detected on-chip (AOC_FSC_ of 0° and AOC_SSC_ of 50°), (**c**) the SSC P2B versus FSC P2B scatter plot of a mixture of 3 µm, 6 µm, and 10 µm beads measured on-chip (AOC_FSC_ of 0° and AOC_SSC_ of 50°) and (**d**) the scatter plot of a mixture of 3 µm, 6 µm and 10 µm beads measured on a benchmark flow cytometer.

The performance of the on-chip flow cytometer with the nominal configuration (AOC_FSC_ of 0° (ALL) and AOC_SSC_ of 50°) was first studied experimentally using polystyrene beads measuring 3 µm, 6 µm, and 10 µm in diameter. The scattering from these beads is relatively strong and helps to understand the system performance before characterization with the cells. The scattering signals are characterized using the metrics peak-to-baseline (P2B) amplitude and the area. P2B is the absolute value of the difference between the maximum or minimum (in the case of FSC ALL) and the baseline of the signal, while the area is calculated as the integrated deviation from the baseline. In this configuration, the FSC ALL is produced by the particle blocking the illumination beam and hence produces a dip in the signal. The amplitude of the dip is mainly determined by the particle size and allows for clear discrimination between the three types of beads. For SSC, the amplitudes of the scattering signal also increase with increasing particle size. Yet, there is a significant overlap between the 3 µm and 6 µm beads and between the 6 µm and 10 µm bead populations. This can be seen from the histograms and scatter plot in Fig. 3.

**Figure 4:**
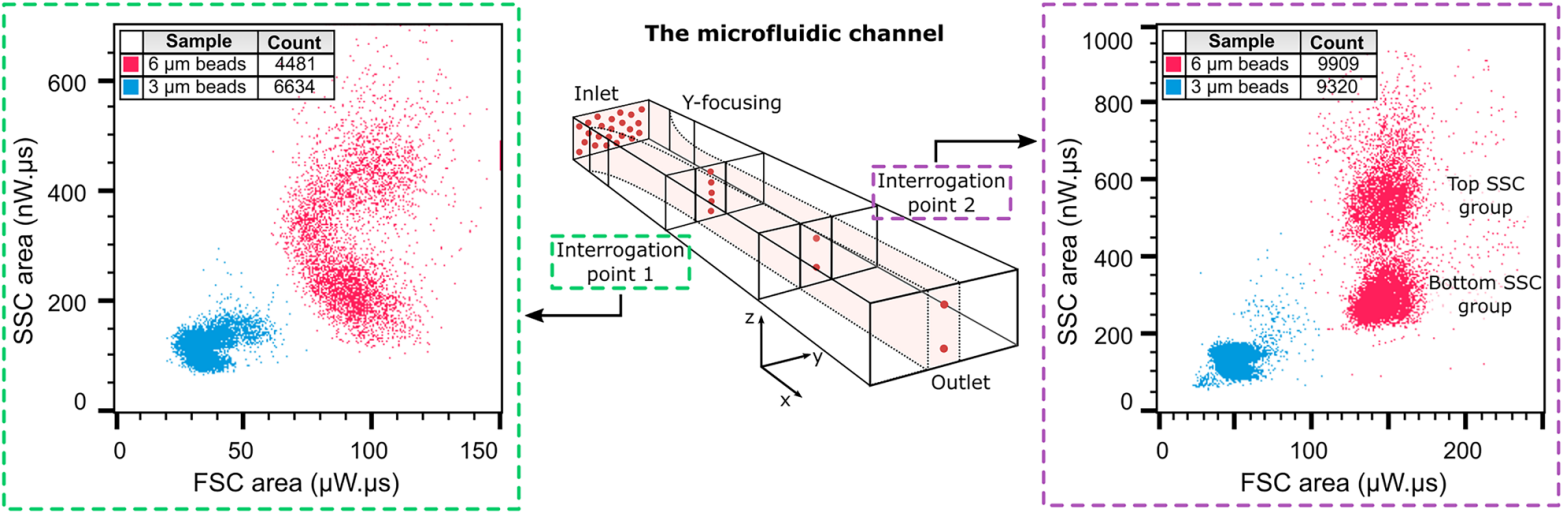
Impact of fluidic Z-focus on the bead scatter plots**.** Schematic drawing of the inertial focusing taking place in the microfluidic channel, showing the distribution of beads at the inlet and two identical optical interrogation points (AOC_FSC_ of 0° and AOC_SSC_ of 50°) separated by 0.5 cm. For each optical interrogation point, the scatter plot of the SSC area vs. FSC area as measured for 3 µm and 6 µm polystyrene beads is shown. The difference in the amplitudes is attributed to the difference in illumination power which amounted to 3.3 mW and 4.2 mW for interrogation point 1 and 2, respectively.

The three FSC populations, clearly distinguishable on the histogram in Fig. 3**a**, have small coefficients of variation (CV)—14.3%, 9.1%, and 3.5% for 3 µm, 6 µm, and 10 µm beads, respectively, as determined using Flowjo™ (see Materials and Methods). The FSC discrimination factor, given by the ratio of the population medians, amounts to 3.3 for the 3 µm and 6 µm beads and to 1.9 for the 6 µm and 10 µm beads. For SSC, each bead population consists of 2 groups. When only considering the bottom groups, the discriminating ratio amounts to 2.7 for the 3 µm and 6 µm beads and 1.8 for the 6 µm and 10 µm beads. For the top groups, the discriminating factors are slightly larger— 3.6 and 2.0, respectively. The two groups within the SSC can be explained by the sensitivity of the SSC collection to the Z-position of the particles in the microfluidic channel. This is unlike FSC ALL, which is produced by the particle blocking the illumination beam and consequentially does not depend on its Z-position. For SSC, a positional offset towards the bottom/top of the channel changes the angular distribution of the scattered light in the plane of the SSC collection gratings. As a consequence, for different Z-positions, a different portion of scattered light is collected by the SSC gratings. In our device, the particles are initially distributed along the entire height of the channel and while flowing down the channel assume equilibrium positions near the top and bottom channel walls due to a fluidic principle called inertial focusing^18,27^. To verify this assumption, we include the results for 3 µm and 6 µm polystyrene beads obtained on a second interrogation point, positioned closer to the fluidic inlets in Fig. 4. As expected, the figure shows that the two SSC groups become smeared out closer to the fluidic inlets. A more detailed discussion on inertial focusing can be found in the SI.

Finally, the polarization of the light detected at the edge coupler was measured to study whether adding a polarizer at the output, selecting either vertical (TE) or horizontal (TM) polarization, can increase the SNR (see Materials and Methods). The FSC signal is a result of the attenuation of the TE-polarized illumination beam and hence maintains the polarization of the baseline light. The P2B amplitude and area of the TM component in the pulses were weaker by at least two orders of magnitude. In contrast to FSC, SSC has a notable TM component with a similar P2B amplitude compared to the TE component but with lower background and RMS noise power (see Table S1). This results in a higher SNR for TM-polarized light, 250 for TM compared to 120 for TE as measured for 6 µm beads. Hence, adding a polarizer at the SSC output has the potential to improve the SSC detection limit twofold.

### Detection of isolated PBMC populations

For use in cell therapy, the on-chip flow cytometer must be capable of discriminating the two main populations in a PBMC sample, lymphocytes and monocytes. In this section, the discrimination potential is studied using isolated monocyte and lymphocyte populations. The scattering pulses generated by cells are characterized using the metrics peak-to-peak (P2P) amplitude and area. The former is calculated as the difference between the maximum and the minimum signal value. The latter represents the total integrated area of both positive and negative contributions to the signal.

**Figure 5:**
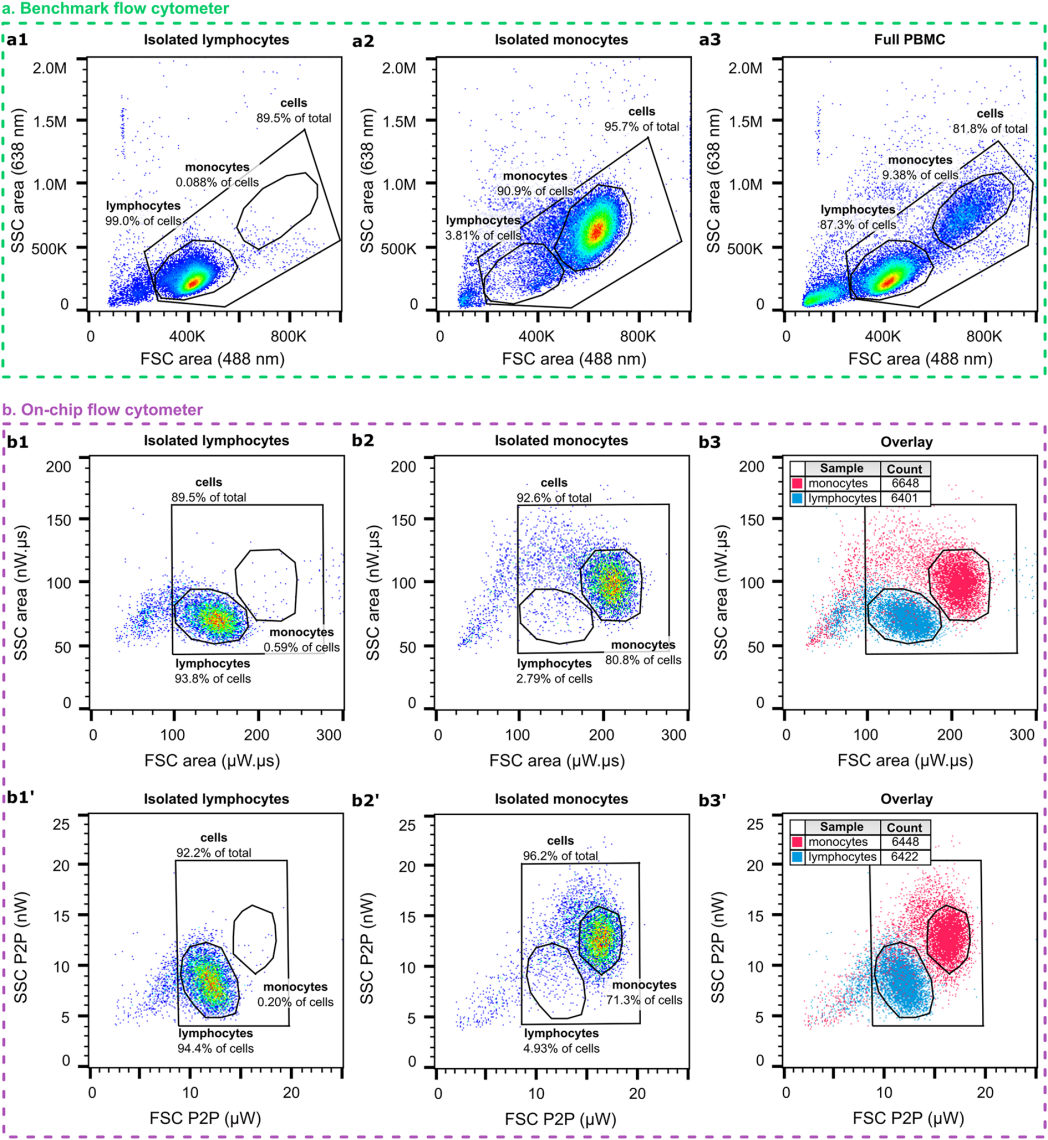
Scatter plots of the (isolated) PBMC populations on-chip and benchmark with a commercial flow cytometer. (**a**) Benchmark flow cytometer scatter plots of (**a1**) isolated lymphocytes (54,517 events), (**a2**) isolated monocytes (55,058 events), and (**a3**) the complete PBMC sample before isolation (60,988 events). **b**) On-chip flow cytometer (AOC_FSC_ of 0° and AOC_SSC_ of 50°) SSC P2P vs. FSC P2P and SSC area vs. FSC area scatter plots of (**b1, b1’**) isolated lymphocytes, (**b2, b2’**) isolated monocytes, and (**b3, b3’**) the overlay of both isolated populations. Here, the total event count for both isolated populations is indicated in the figure legends.

To compare the performance of the on-chip flow cytometer with a commercial tool, the biological sample is first characterized on our benchmark flow cytometer (CytoFLEX). Figure 5**a** shows the scatter plots for both the isolated populations (panels **a1** and **a2**) and the complete PBMC sample before isolation (panel **a3**). The corresponding on-chip-recorded SSC versus FSC scatter plots for the isolated lymphocyte and monocyte populations are presented in panels **b1–b2** for the area and in panels **b1’–b2’** for the P2P amplitude.

For lymphocytes, the cell type producing the smallest signal, the on-chip-recorded SNR amounts to roughly 51 and 18 for FSC and SSC, respectively. For monocytes, the SNR measures 66 and 27, for FSC and SSC, respectively. Yet, unlike for the bead measurements, there was no obvious advantage in adding a polarizer at the output to select either vertical or horizontal (TM or TE) polarization. The FSC ALL signal holds mainly the TE-polarization of the illumination light with a minimal contribution (~ 1%) of TM-light. For SSC, the RMS noise amplitude for TM polarization is half of that of TE, but the signal from cells is also lower in TM polarization, on average by a factor of 2. This results in a similar SNR for both polarizations. Table S2 in the SI summarizes the median P2P amplitude and area measured on-chip for the isolated cell populations shown in Fig. 5.

The on-chip detection can discriminate the isolated lymphocyte and monocyte cell populations as evidenced by the overlay scatter plots in panels (**b3**, **b3’**) and the purities of the gated fractions. Less than 1% of the isolated lymphocyte sample overlaps with the monocyte gate (panels **b1**, **b1’**) and less than 5% of the isolated monocyte sample ends up in the lymphocyte gate (panels **b2**, **b2’**). These purities are very similar to those measured on the benchmark flow cytometer. In fact, the on-chip discrimination factor of FSC in ALL mode amounts to 1.4, which is on par with the discriminating factor of 1.4 obtained on the benchmark flow cytometer. Also, the population CVs are comparable. For the benchmark flow cytometer, the CVs in FSC amount to 6.7% and 6.6% for lymphocytes and monocytes respectively, when discriminated on peak amplitude. On-chip the corresponding CVs amount to 10.8% and 11.9% for discrimination on P2P amplitude. For SSC, the on-chip discrimination factor on P2P amounts to 1.5 with population CVs of 19.2% and 15.0% for lymphocytes and monocytes, respectively, compared to a discrimination factor of 2.5, with population CVs of 22.7% and 16.8%, on the benchmark. The on-chip SSC discrimination can likely still be improved upon the implementation of Z-focusing ^28^ since it was found for the polystyrene beads that the collected scattering heavily depends on the Z-position of the particle. All the discrimination factors for FSC and SSC on-chip and on the benchmark flow cytometer are summarized in Table S3 in the SI.

### Detection of full PBMC populations

**Figure 6:**
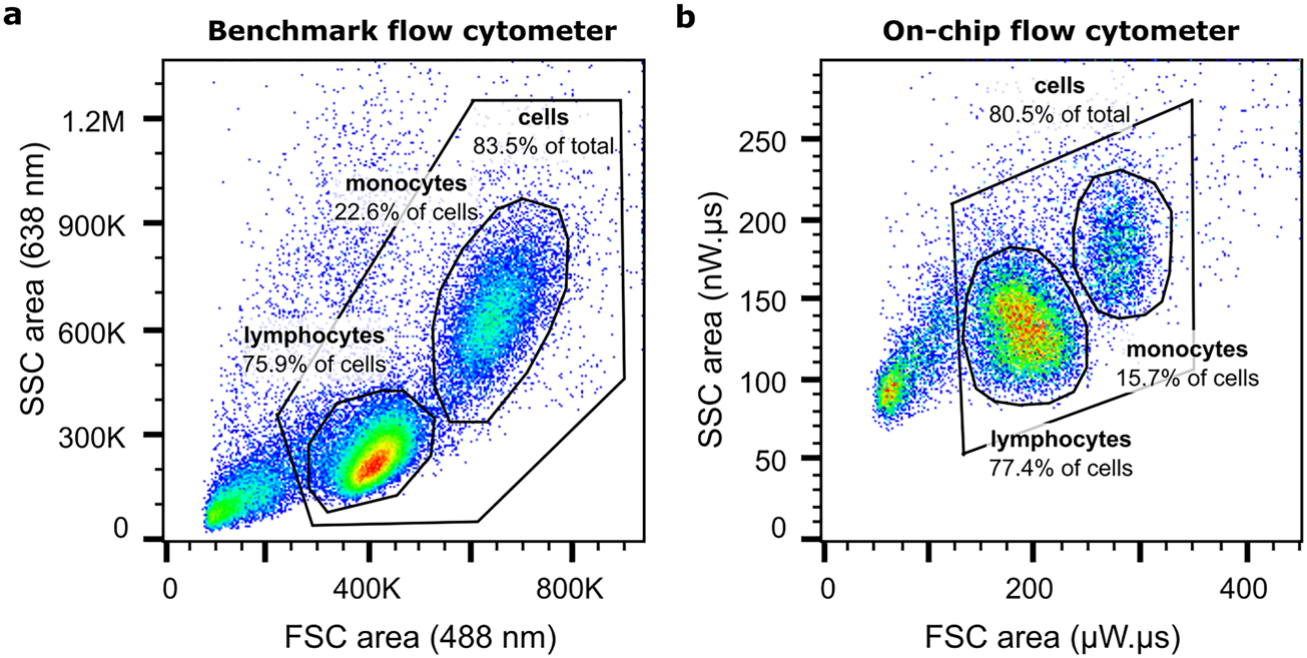
Full PBMC scatter plots on-chip and on a benchmark flow cytometer. Scatter plots of a full PBMC sample measured on (**a**) a benchmark flow cytometer (60,084 events) and (**b**) the on-chip flow cytometer (AOC_FSC_ of 0° and AOC_SSC_ of 50°) (19,168 events). For each scatter plot, the total cell population is gated, as well as the monocyte and lymphocyte fractions therein.

Figure 6 presents the SSC area versus FSC area scatter plots for a complete PBMC sample run through the benchmark flow cytometer in panel **a**, and the on-chip flow cytometer in panel **b**. The on-chip flow cytometer allows for discrimination of the monocyte population from the lymphocyte population as well as smaller debris present in the sample. Based on the gating shown in Fig. 6, the composition of the cell population as found on-chip, 15.7% monocytes, and 77.4% lymphocytes, corresponds well to the composition given by the benchmark flow cytometer, 22.6% and 75.9%. On-chip, the monocyte and lymphocyte populations can be classified with a discrimination factor of (1.51 ± 0.12) for FSC area and (1.36 ± 0.05) for SSC area (averaged over three repeats). Moreover, the on-chip cytometer is capable of detecting debris at low nW light levels. This gives confidence that the complete cell population is detected and represented on the scatter plot, as well as extra in-line information on the quality of the input sample.

### The impact of the angle of collection

Apart from the characterization of the nominal configuration design (with AOC_FSC_ 0°(ALL) and AOC_SSC_ 50°), presented above, also other collection grating configurations were characterized. Forward scattering was detected not only in ALL mode but also in scattering mode, with the FSC gratings collecting scattered light at an AOC_FSC_ at 5°. In this configuration, the background was lower by two orders of magnitude, the event pulses were positive, and the signal with a power of a few µW was strong enough to be detected by silicon photodiodes. In this configuration, two FSC populations per cell type (or bead size) were detected, meaning the forward scattered light is sensitive to the Z-position of the particles, similar to what was found for the SSC (see Figure S3).

Side scattering was also measured on structures designed for an AOC_SSC_ of 70°. This configuration allowed for a lower background than in the design with an AOC_SSC_ of 50°, but the signal was also lower by approximately a factor of 5 in area and in P2P amplitudes. This matched the expectations because it is known that the intensity of scattering reduces with increasing angle. Yet, the SSC discrimination potential increases with increasing AOC. We measured a discrimination factor of 1.2, 1.5, and 1.6 for an AOC_SSC_ of 40°, 50° and 70°, respectively. The scatter plots for optical detection devices with an AOC_SSC_ increasing from 40° to 50° and finally, 70° can be found in the SI, Figure S4.

### Fluorescence

**Figure 7:**
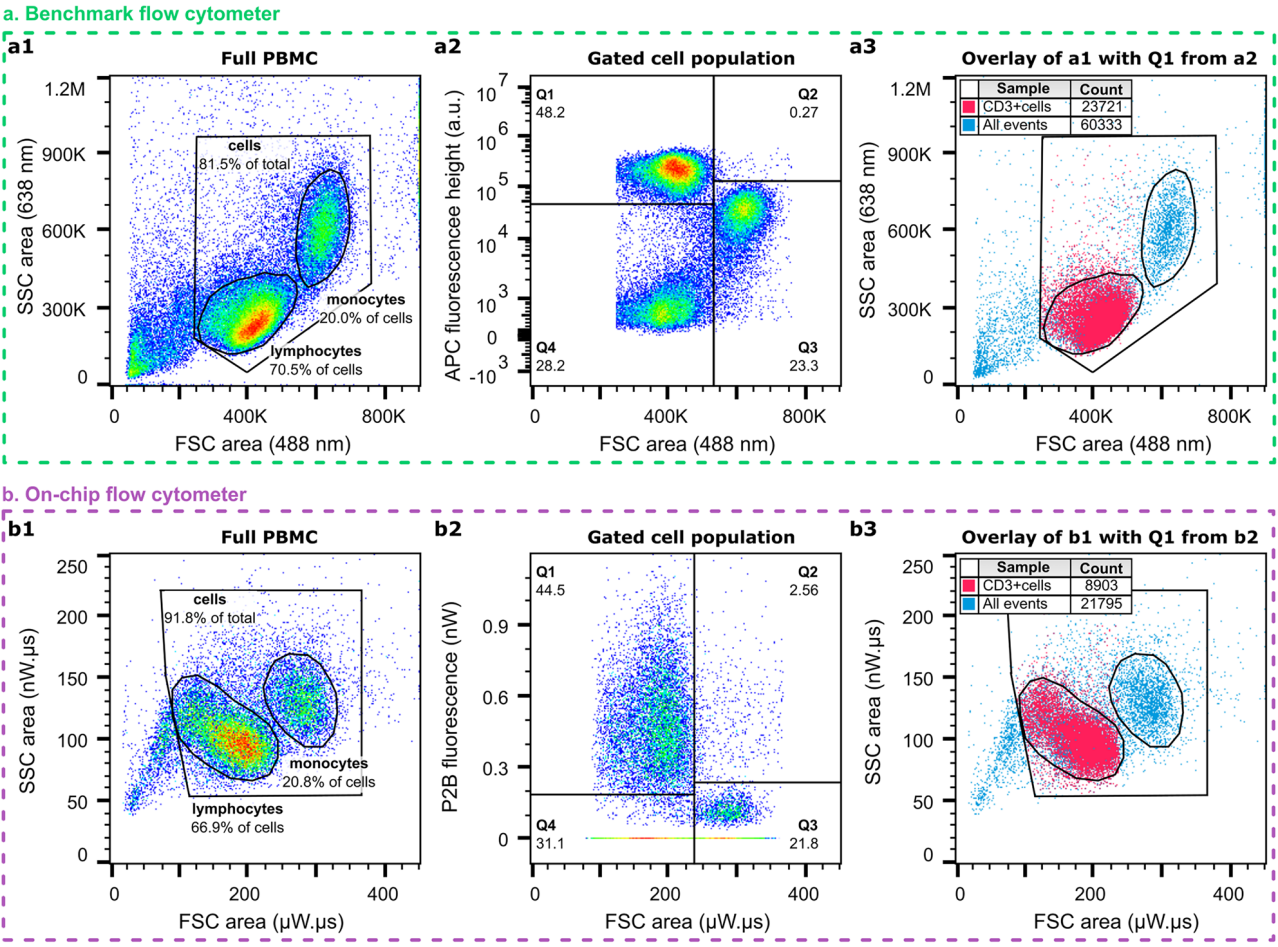
Detection of fluorescence anti-CD3-APC markers on-chip and on a benchmark flow cytometer. (**a**) Benchmark flow cytometer scatter plots and (**b**) On-chip flow cytometer scatter plots of the same PBMC sample with fluorescent CD3-APC labeling. (**a1, b1**) the SSC area vs. FSC area scatter plot with gating of the total cell population as well as the monocytes and lymphocytes therein, (**a2, b2**) APC fluorescence vs. FSC area scatter plot of the total cell population as gated in panels (a1, b1), and (**a3, b3**) overlay of the CD3-positive population (Q1 in panels a2, b2) on the scatter plot from panels (a1, b1). Here, the total event count as well as the number of CD3 +  cells from Q1 in panels (a2), and (b2﻿) is indicated in the figure legends.

Besides collecting forward and side scatter with integrated optics, the chip architecture also allows the measurement of fluorescence excited by the integrated on-chip illumination using a fiber mounted above the chip. The system capabilities were tested using Quantum™ MESF beads and validated with immunofluorescence staining of PBMC samples. The sensitivity of the system was enough to detect APC beads with an MESF value of 1,200,523 with an SNR of 9 and PBMC samples in which either lymphocytes were stained with anti-CD3-APC or monocytes with anti-CD14-APC, with an SNR of 20. The sensitivity was limited by background light, the origin of which is being investigated. Figure 7 shows a comparison of the detection of a full PBMC sample with an anti-CD3-APC labeled lymphocyte population. There is a good agreement between the fluorescent lymphocyte count, 48.2% for the benchmark flow cytometer (The top left quadrant (Q1) in panel **a2**) and 44.5% for the on-chip flow cytometer (Q1 in panel **b2**). In the latter measurement, events for which FSC and SSC but not fluorescence was detected, a fluorescence value of zero was assigned during data post-processing. Backgating the fluorescent population onto the scatter plot in panels **a1, b1** allows for the validation of the discrimination based on light scattering. The overlay of the scatter plot with the CD3 + lymphocyte population is shown in panels **a3**, **b3**. On-chip, 95.3% of the CD3 + cells within Q1 fall within the lymphocyte gate, compared to 94.5% on the benchmark flow cytometer. An overview of the accuracy of the cell classification can be found in Table S4.

## Conclusions and perspectives

We have fabricated an on-chip flow cytometer for the in-line classification of different cell populations based on forward- and side-scattering characteristics. Fluidics, illumination-, and collection optics for forward- and side-scattered light were monolithically integrated. Both forward and side scattering from cells could be reliably detected using integrated optics and the device performance was validated for the classification of lymphocytes and monocytes. In fact, the nominal configuration design allowed for the discrimination of monocytes, lymphocytes, and debris in a complete PBMC sample with a monocyte/lymphocyte discrimination factor of 1.5 on FSC and 1.4 on SSC. This is for FSC on par with the performance of our benchmark flow cytometer. For SSC, the discrimination is somewhat lower on-chip, although it was shown to improve with increasing AOC. Moreover, the design has been successfully shown to allow for the collection of fluorescence through the transparent quartz top wafer.

The forward scattering, both ALL at 0° and scattering at 5°, was detected using PMTs but is strong enough to be detected by silicon photodiodes. The SSC is weaker and requires more sensitive detectors (PMT, APD) and low-noise readout electronics. The collection efficiency of the SSC gratings and FSC in scattering mode (5°) is lower than in systems using external optics, where the scattered light can be collected from apertures with large solid angles. At first glance, light from a broad range of incident angles might be collected on-chip as well using a larger array of collection gratings (i.e. increasing the number of gratings). In practice, the complexity and geometrical constraints related to the routing of the waveguides to the gratings, the tight geometry at the interrogation point, the proximity of the illumination grating, as well as light scattering on fabrication imperfections, do not allow to allocate collection gratings everywhere. This limitation, in combination with the limited efficiency of the coupling elements, does not permit collection efficiencies to be as high as in the systems with bulk optics. This can be translated to a fundamentally lower sensitivity of devices on-chip. Moreover, the collection of scattered light (SSC, and FSC at 5°) also suffers from measurement variability due to the spread of particle positions along the height of the microfluidic channel. The implementation of fluidic Z-focusing ^28^ in combination with optimized collection gratings (for collection efficiency) and illumination gratings (for higher uniformity of the illumination intensity in the center of the microfluidic channel, as well as to provide better directionality of the illumination beam) is expected to further reduce the spread (i.e. the CVs) of the bead and cell populations and to bring it closer to the benchmark values. Moreover, detectors with higher quantum efficiency can be considered for future improvements in the setup.

Despite the above-mentioned constraints, we were able to robustly detect and reproduce the FSC/SSC profile of PBMCs. The results presented in this work were performed using sample concentrations of 10^6^ cells/ml at flow rates of 100 µl/min (with a sample flow rate of 10 µl/min), which corresponds to detection rates of roughly 10,000 cells/min and a particle flow speed of approximately 0.5 m/s. To achieve higher cell throughput per channel, the flow rate and thus the acquisition speed can be proportionally increased. This requires broadening the system bandwidth and reducing the integration time per sample to preserve the information in the scattering pulse shapes with the same resolution. Additionally, to maintain the SNR and resolution, more illumination power would be required. The typical duration of the discussed experiments ranges from 30 s to 3 min. There is no fundamental reason to expect a compromised system stability during longer experiments. The operation of the on-chip photonic components is alignment-free and potential mechanical drift of the in-coupling fiber with respect to the in-coupling grating, which could cause a drop in the excitation light intensity, was compensated by the grating design. The system performance was also verified experimentally with higher sample concentrations (up to 10^7^ cells/ml) and longer experiment duration (up to 30 min). During these experiments, the flow and pressure were continuously monitored and no flow instabilities were observed. Yet, the main advantage of our on-chip flow cytometer lies in its potential for parallelization of multiple flow channels to increase the system throughput. Based on our proof-of-concept, the potential dimensions of a single fluidic channel including a bubblejet sorter ^24^ with input and output, an interrogation point for FSC and SSC, waveguide routing, and out-coupling of the light can be reduced to at least 2 × 8 mm. In combination with its alignment-free operation at the interrogation point, the on-chip flow cytometer can then be designed as a GMP-compliant, closed-loop, disposable, multichannel device. Together with data analysis automation, the system could be operated with minimal human intervention which allows for the simultaneous operation of multiple devices in parallel without risking cross-sample contamination.

Our successful implementation of cell analysis on-chip is a first step towards the technological development of miniaturized flow cytometry devices. Next, the co-integration of a cell sorting module^24^ followed by the integration of detectors and front-end electronics, all using monolithic or hybrid technologies, will enable further developments. Herein, the optics on-chip allows for design flexibility. The photonic components and the layout can be tailored to address the needs of specific applications. For example, if the collection of scattered light from one or another point can provide relevant information, the position of the collection gratings can be optimized accordingly. The flexibility offered by photonics opens new frontiers in the design of miniaturized cytometry systems and integration in fully automated workflows. Additionally, various illumination colors can be implemented for light-scattering and the excitation of fluorescence. SiN photonic platforms support wavelengths throughout the visible and NIR spectral range such that the operational wavelength can be easily adapted. As the components are small, there could also be more than one interrogation point for either FSC, SSC, or fluorescence allocated within a compact area to allow for in-line quality control of both sorted and waste fractions.

## Materials and methods

### Chip fabrication

The chip was fabricated in imec’s 200 mm CMOS Pilot Line and consists of a stack of three bonded wafers: (1) a bottom silicon wafer with the illumination photonics, (2) a middle silicon wafer containing the fluidics, and (3) a top quartz wafer with the collection photonics. The photonic circuits on both the illumination (bottom silicon) and the collection layer (top quartz) were fabricated using Imec’s 180 nm PECVD (plasma enhanced chemical vapor deposition) silicon nitride (SiN) platform^29^. Specifically, the illumination photonic circuits were built on a 725 µm thick silicon wafer. First, 1.9 µm of CVD (chemical vapor deposition) oxide was deposited on the bare silicon wafer, followed by the deposition and patterning of a 100 nm thin Al layer as a reflector below the photonic grating structures. Then, a high-density plasma CVD oxide was deposited and polished using chemical mechanical polishing (CMP) to obtain a target oxide thickness of 420 nm. On top, the 180 nm SiN layer was patterned using 193 nm optical lithography and a reactive ion etch process. Thereafter, a high-density plasma CVD oxide was deposited as the top cladding and the surface was polished by CMP to reach a target of 2.3 µm. Finally, the fluidic inlets and edge coupler areas were patterned using a deep oxide and silicon etch process to etch 80 µm into the silicon substrate.

The collection photonic circuit was built on a 725 µm thick quartz wafer. First, 100 nm of CVD oxide was deposited. On top of the oxide, a thin layer of 140 nm titanium nitride (TiN) was then deposited and patterned on the part of the wafer that was free of photonics to absorb unwanted light, that could be scattered by the photonic circuits and propagate along the slab layers (SiO_2_ cladding and quartz substrate of the top wafer) to the edges. After the patterning of the 180 nm SiN photonic circuits, a CVD oxide was deposited as the top cladding and polished using CMP to obtain a target thickness of 2.3 µm.

To fabricate the fluidics layer, a thin layer of 100 nm thermal oxide was grown on a 725 µm silicon wafer. This wafer was then bonded (unaligned) to the quartz wafer with the collection photonics by fusion bonding. The fluidic circuits wafer was subsequently ground and polished to a thickness of 30 µm, followed by deposition of a layer of low-temperature oxide and polishing of the oxide surface to reduce roughness and to obtain a thickness of 150 nm. Then, the fluidic channels were patterned onto the wafer using a deep silicon etch process. Thereafter, the wafer containing the illumination photonics was bonded to the fluidic-quartz wafer stack by aligned fusion bonding at the wafer level. Our wafer-scale bonding achieved a bonding accuracy of < 5 µm, which is expected to further improve to less than 200 nm soon. An important step forward and technological novelty was the successful bonding of 200 mm wafers, which enables high-volume manufacturing when compared to die-to-die bonding. Finally, the illumination photonic circuit wafer was ground to a thickness of 200 µm, the fluidic inlets were patterned using a deep silicon etch process, and the wafer was diced into chips.

### Experimental setup

#### Fluidics

For testing, the chip is clamped into a holder. Fluidic connection is provided by ferrules (N-123-03X, IDEX Health and Science LLC) and tubing with a 150 µm inner diameter and 360 µm outer diameter matching the positions of the inlets of the microfluidic channels. 1/32″ outer diameteter PEEK™ tubing (IDEX Health and Science LLC) is used to connect the inlets with 3 Legato 110 syringe pumps (KD Scientific, Holliston USA). Pressure sensors and flow meters are inserted in the fluidic tubing to monitor the flow rates and flow stability. For both sheath flows a 10 ml syringe (BD Plastipak) is used and a unit M flow meter (Fluigent) is inserted in the microfluidic path. For the sample flow, a 1 ml syringe (BD Plastipak) is used, and a pressure sensor (Labsmith, uPS0800) is inserted in the fluidic path. Finally, the waste flow is connected to a unit L flow meter (Fluigent). All the experiments were performed at a flow rate of 100 µl/min, corresponding to an average particle speed of 0.5 m/s, with a sample-to-sheath ratio of 1:9 (2 × 45 µl/min sheath flow rate, 10 µl/min sample flow rate). This sample-to-sheath ratio allowed for hydrodynamic focusing along the Y-axis with a sample stream width of roughly 10 µm (FWHM). In the Z-direction, no flow focusing was applied.

#### Optics and readout electronics

**Figure 8:**
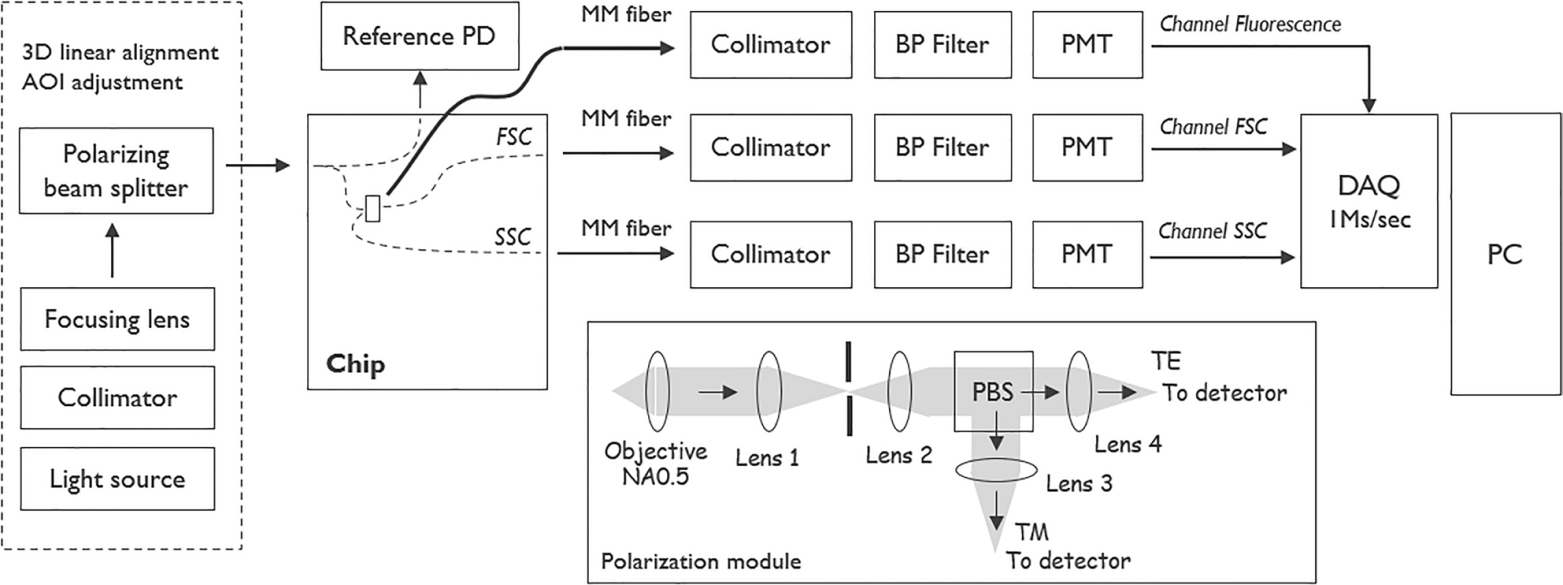
Block diagram of the experimental setup. Block diagram showing the in-coupling optics, the chip, the optics for collecting the output light, and the readout electronics. The module with external optics used for measurements of the polarization of the output light is shown in the inset.

The block diagram of the experimental setup is presented in Fig. 8. Specifically, a polarization-maintaining fiber from a pigtailed laser source (OBIS-LX-FP-637 100 mW, Coherent, Inc.) was connected to a collimator (Thorlabs, TC06APC-633). The collimated light was projected onto a doublet achromatic lens (Thorlabs, APC125-75) and then directed to a polarizing beam splitter (Thorlabs, PBS051) which reflected the TE-polarized component towards the chip. To maximize the power of the incident light, the collimator with the fiber was rotated to match the polarization of the collimated beam to the polarization of the light reflected by the polarizing beam splitter. A reference channel was added on-chip to facilitate the alignment of the excitation beam to the in-coupling grating. The output of the channel was monitored with a reference photodetector (PD): a silicon detector connected to a trans-impedance amplifier.

The FSC and SSC output light was collected using multimode (MM) fibers with a core diameter of 400 µm and an NA of 0.39. After the fibers, the light was collimated (FC230SMA) and sent to photomultiplier tubes (PMT, Hamamatsu H10723-01) through optical bandpass filters (BP filters Thorlabs FLH640/10). The BP filters were used to filter out ambient light. Fluorescence was collected from above the chip using a MM fiber (400 µm diameter and an NA of 0.39), tilted 40° with respect to the chip normal to avoid direct excitation light in the collection path. The collected light was further filtered by two optical bandpass filters (Semrock FF01-670/30-25). The PMTs were connected to an acquisition card (National Instruments, USB NI-7855R) measuring the voltage at a sampling rate of 1 MS/s and with a bit depth of 16 bits per channel. Three PMTs were installed for the simultaneous detection of FSC, SSC, and fluorescence signals. The PMTs were calibrated in-house to correlate the incident power to the counts generated by the acquisition card.

To measure the polarization of light coming out of the FSC/SSC channels, a projection system with a polarizing beam splitter (see inset in Fig. 8) was used. The light from the waveguide facets was collected using an objective lens with NA 0.5 and then projected onto a slit with a tube lens so that the light that did not originate from the edge couplers was blocked and the light coming from the waveguides was projected onto the detectors by another lens pair after going through a polarizing beam splitter (PBS).

The volumetric intensity distributions in Fig. 2**a** were constructed from an image stack measured using a microscope equipped with a motorized stage, a water-immersion objective with a magnification of 60×, and a CMOS camera.

### Calibration particles

The FSC and SSC detection capabilities of the interrogation points on-chip were characterized using a micron bead calibration kit containing 3 µm, and 6 µm diameter polystyrene beads (Bangs Laboratories, Inc. Fishers US) in combination with 10 µm diameter Envy-green fluorescent beads (Bangs laboratories). The capability of detecting fluorescence with on-chip illumination was studied using a Quantum™ MESF APC calibration kit (Bangs Laboratories). All the samples were diluted to a concentration of 10^6^ particles/ml, resulting in detection rates of 150 events per second.

### Cell preparation

The white blood cells are obtained from the Red Cross biobank and further processed in the lab to procure a library of peripheral-blood-mononuclear-cell (PBMC) aliquots from one donor. The protocols for cell isolation and fluorescence staining can be found in the SI.

### Benchmark cytometry measurements

Every sample was measured in parallel on the CytoFLEX S as a benchmark. The detector configuration was set as follows: FSC was collected per default on the 488 nm laser, but an extra 638/8 nm filter was introduced in the 638 nm laser emission path to collect SSC from the 638 nm laser. For fluorescence collection of APC, a 660/10 bandpass filter was used.

### Data processing

Raw data was processed using Anaconda for Python 3.8. A complete description of the processing algorithm can be found in the SI. Data visualization was performed using Python and FlowJo™ (BD Sciences), a commercial flow cytometry software package.

Analysis of the cell populations was performed in Flowjo. First, the cells were gated based on the forward- and side-scattering to remove cell debris and events that could represent more than one cell. Next, the lymphocyte and monocyte subsets were gated from the resulting cell population using the SSC area versus FSC area or SSC P2P versus FSC P2P scatter plots. In the figures, both the cell gate and the lymphocyte/monocyte subgates are overlayed on the scatter plot of the original ungated measurement. Only in Fig. 7**b**, the fluorescence is shown for the gated cell population and the CD3-positive fraction therein is backgated onto the original scatter plot in panel **c** of the same figure.

### Modeling

The commercial FDTD tool Lumerical was used for simulating the scattering profile of a 6 µm bead in Fig. 2**b**.

### Supplementary Information

Supplementary Information.

## Data Availability

The generated data sets are available from the corresponding author on reasonable request.
