# Supplementary material for: On-chip flow cytometer using integrated photonics for the detection of human leukocytes
Source: Sci Rep. 2024 May 20;14:10921. doi: 10.1038/s41598-024-60708-0 (PMC11106258; doi:10.1038/s41598-024-60708-0)
Supplement: Supplementary file 1 — Supplementary Information. [file 41598_2024_60708_MOESM1_ESM.pdf]

Supplementary information for

On-chip flow cytometer using integrated photonics for the detection of human leukocytes.

Stijn Jooken[1,†,*], Kirill Zinoviev[1,†], Günay Yurtsever[1], Anabel De Proft[1], Koen de Wijs[1], Zeinab Jafari[1], Ana Lebanov[1], Gaudhaman Jeevanandam[1], Mateusz Kotyrba[2], Erwin Gorjup[2], Jelle Fondu[1], Liesbet Lagae[1], Sarah Libbrecht[1], Pol Van Dorpe[1], Niels Verellen[1,*].

[1] *imec, Kapeldreef 75, 3001, Leuven, Belgium*
[2] *Sarcura GmbH, Plöcking 2,3400, Klosterneuburg, Austria*
† *These authors contributed equally*
* *Corresponding authors*

Preparation of plain PBMC from buffy coat.

A buffy coat was obtained from the Red Cross and further processed in the lab to procure a library of PBMC aliquots from one donor. First, the blood was diluted 1:1 in PBS (phosphate buffered saline) and 30-35 ml of the blood was layered on 20 ml Ficoll-Paque (GE17-1440-02, Merck). After gradient centrifugation (800 × g for 20 min without brakes), the plasma was removed, and the mononuclear layer was collected. After three wash cycles with PBS (centrifugations at 300 × g for 10 min), the PBMC were resuspended at 60 million/ml in pure FBS (fetal bovine serum). Next, the same volume of 2X concentrated freezing medium (20% DMSO in FBS) was slowly added. The solution was then divided into cryovials and transferred to the -80 °C overnight before long-term storage in the liquid nitrogen tank.

Preparation of measurement sample from frozen PBMC aliquot.

On the day of the measurement, a PBMC aliquot was thawed, diluted with warm RPMI medium, centrifuged (400 × g, 5 min), and resuspended in MACS buffer (2 mM EDTA, 0.5% BSA, PBS). The cells were counted, and the concentration was adjusted based on the experiment to be performed. When a pure PBMC sample was used for optical measurements, the concentration was reconstituted to 1 million/ml in MACS buffer and filtered through a 30 μm mesh filter before loading into the syringes.

For the optical measurement of isolated lymphocytes or monocytes, a magnetic bead isolation was performed per manufacturer's instructions (Pan T cell isolation kit, 130-096-535, Miltenyi and a classical monocyte isolation kit, 130-177-337, Miltenyi). After isolation, the individual cell populations were counted and reconstituted to 1 million/ml in MACS buffer.
For immunofluorescence measurements, cell staining was performed on the pure PBMC sample. The sample was split into 3 fractions to obtain an unstained control, a CD3-APC stained sample and a CD14-APC stained sample. A maximum of 6 million cells was resuspended in 100 μl MACS and 5 μl of CD3-APC (Biolegend, 300312) or CD14-APC (301808) was added. After incubation on ice for 20 minutes, the samples were washed twice with MACS (300 × g, 5 min) and resuspended in 1 million/ml MACS buffer.

For all experiments, an aliquot of the sample was measured on the CytoFLEX S as a control and benchmark readout.

Data processing.
After recording, the raw electrical signal data from the PMT modules were processed offline into meaningful metrics and statistics to evaluate the performance of the devices. All offline data processing was done using custom Python code and can be split into three main parts, (1) data cleaning, (2) event detection and characterization, (3) and compilation of the event characteristics into meaningful statistics. In the first part, data cleaning, the raw PMT counts were converted into power (expressed in nanowatts) using the gain setting and calibration of the PMT. In addition, the baseline wander (which might originate from different causes like ambient light changes, laser fluctuations, or small mechanical drifts in the measurement setup), was removed with a smoothing algorithm[1].

Secondly, events were detected and characterized for both the FSC, SSC and (optionally) fluorescence traces separately. The word "event" covers the optical signature of any particle passing through the microfluidic channel. Before event detection, the raw signal was passed through a 5th-order Butterworth filter with a critical frequency of 0.1 MHz. On this filtered raw signal, the parameters for event detection were extracted: the RMS noise and the baseline. Events were then detected with the scipy.signal.peak_finder algorithm, with the minimum temporal spacing between two peaks set to 40 µs, the peak threshold set to 5 times the noise standard deviation, and the minimum peak width set to 5 µs.

After event detection, the waveforms were characterized on the raw data trace according to three metrics—the peak-to-baseline (P2B) amplitude, the peak-to-peak (P2P) amplitude, and the area under the curve. The peak-to-baseline amplitude is calculated as the absolute value of the difference between the maximum of the detected peak and the baseline. The peak-to-peak amplitude was calculated as the difference between the maximum and minimum value of the signal within a 50 µs time interval around the peak location (25 µs to the left and 25 µs to the right). Finally, the area was calculated by integrating the absolute value of the signal minus the baseline ($\int |signal - baseline|$) within the same time interval used for the peak-to-peak calculation using the Simpson rule.

Once the events had been detected and characterized for each data trace, the FSC and SSC events were matched. Towards this end, for each event in the FSC trace, a corresponding SSC event was searched that occurred within 100 µs of the FSC event. Because the SNR on the SSC signal is significantly lower than that of the FSC signal, we assume no SSC event can be detected without its corresponding FSC event in the vicinity. If no SSC peak could be matched, the event was categorized as a false-positive and discarded. Then, optionally, to every FSC-SSC pair a fluorescence event was matched in the same way. If no fluorescence was detected within the 100 µs time frame around the FSC event, a fluorescence value of zero was assigned. Finally, the metrics for all matched events were written to an FCS (Flow cytometry standard) file for data visualization with FlowJo™ (BD Biosciences).

Inertial focusing of particles and cells in the microfluidic channel.
The sensitivity of the SSC collection to the Z-position of the polystyrene beads in the channel can be seen by studying two identical on-chip interrogation points, separated by 0.5 cm along a microfluidic channel. Due to a fluidic principle called inertial particle focusing, a combination of shear-induced lift-forces and particle-wall interactions, particles move across streamlines to assume equilibrium positions near the channel walls. In square microchannels, polystyrene microparticles are known to focus in four equilibrium positions near the channel walls. In low aspect ratio channels, such as those present on our chip, the particles will focus in two

equilibrium positions, centered near the top and the bottom of the channel[2,3]. The effect can be observed in Figure 4 where the SSC area versus FSC area of a mixture of 3 µm and 6 µm polystyrene beads is plotted on a scatter plot for both identical interrogation points. As the FSC (in ALL mode) is not sensitive to the particle's Z-position, variations in the FSC area reflect only the duration of the shadowing and hence hold information on the particle's speed. The velocity profile in a fully developed laminar flow is parabolic with the highest speed at the center and the minimum at the channel walls. A shorter FSC event corresponds to a higher flow speed and thus a more central particle position in the channel. For the first interrogation point in Figure 4, the beads are still distributed over the entire height of the channel and the difference in width from the parabolic flow profile can be observed in the scatter plot. At the location of the second interrogation point, the beads have reached their equilibrium positions near the top and the bottom of the channel. Now, two distinct populations can be observed on the scatter plot. Both populations have the same speed, indicated by identical values for the FSC area, but different SSC intensities because for particles closer/further to/from the SSC collection gratings, a different range of scattered SSC signal reaches the grating, and the collection efficiency drops.

Unlike the beads, for white blood cells, the scatter plots do not reveal distinct groups in SSC that can correspond to cells at different z-positions in the microfluidic channel. Most likely, the lower rigidity of the cells reduces the force that directs them toward the equilibrium positions for inertial focusing. Another reason could be that the reduced intensities of the scattered light from PBMCs compared to beads and the random biological variation within a cell sample make it difficult to distinguish groups and result in a single group with a larger coefficient of variation instead.

On-chip FSC and SSC backgrounds (AOC_{FSC} 0° and AOC_{SSC} 50°).

The typical numbers for the backgrounds are presented in relative units in Table S1 in terms of transmittance, which is defined as the ratio of background to illumination power. On average, the background in the SSC channel is lower by 3 orders of magnitude compared to the FSC channel. The difference between the background measured with the fiber and the objective was due to a significant portion of background light, which is collected by the fiber but filtered out by the slit in the polarization module (see Materials and Methods).

Table S1. FSC and SSC backgrounds.
FSC (AOC_{FSC} of 0°) and SSC (AOC_{SSC} of 50°) backgrounds, as measured on one of the tested chips. The numbers in the table are presented in terms of transmittance, which is the ratio of the power of the collected light to illumination power. Objective TE/TM stands for the measurements done with the polarization module. TE stands for the light polarized parallel to the chip plane, TM – perpendicular.

	FSC	SSC
**Fiber**	$2.4 \cdot 10^{-2}$	$1.1 \cdot 10^{-5}$
**Objective TE**	$2.4 \cdot 10^{-2}$	$1.4 \cdot 10^{-6}$
**Objective TM**	$1.6 \cdot 10^{-4}$	$0.3 \cdot 10^{-6}$

The polarization of FSC and SSC output light for polystyrene beads on-chip (AOC_{FSC} 0° and AOC_{SSC} 50°).

Figure S1 shows scatter plots for the TM versus TE polarization of the light exiting the edge couplers measured for 6 µm polystyrene beads. For FSC in panel **a**, the amplitude and area

of the TM component in the pulses were weaker by at least two orders of magnitude. For SSC in panel **b**, two populations can be distinguished as well, due to the moving of the beads to the stable equilibrium positions by inertial focusing. Here, the TM component has a similar P2B amplitude compared to the TE component.

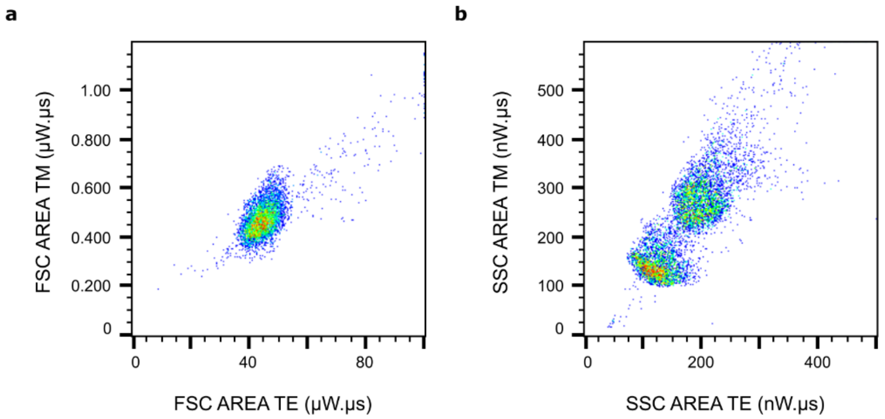

Figure S1. ***Scatter plot of TM vs. TE polarized light measured for 6 µm beads.***

*The scatter plot, built for **a)** FSC and **b)** SSC signals measured at two orthogonal polarizations, TE and TM, using beads with a diameter of 6 µm on the on-chip flow cytometer with an AOC_{FSC} of 0° (ALL) and AOC_{SSC} of 50°.*

Median P2P amplitude and area measured on-chip (AOC_{FSC} 0° and AOC_{SSC} 50°) for isolated cell populations.

Table S2 summarizes the median P2P amplitudes and areas, as well as the robust CV recorded for isolated lymphocyte and monocyte populations shown in Figure 5. The P2P amplitudes and areas are presented as an excitation collection factor (ECF) value, i.e. normalized by the excitation power. The CVs are calculated using Flowjo based on the gating shown in Figure 5.

Table S2. ***Cell population medians and CVs measured on-chip.***

Excitation collection factor (ECF) of the FSC and SSC cell population median measured on-chip (AOC_{FSC} of 0° and AOC_{SSC} of 50°). The ECF is calculated as the signal power normalized to the illumination power.

		FSC		SSC	
**Cell type**	**Metric**	**ECF**	**CV (%)**	**ECF**	**CV (%)**
**Monocytes**	**P2P amplitude**	$5.9 \cdot 10^{-3}$	10.8	$4.7 \cdot 10^{-6}$	15.9
	**area**	$5.0 \cdot 10^{-3}$	16.3	$2.5 \cdot 10^{-6}$	15
**Lymphocytes**	**P2P amplitude**	$4.4 \cdot 10^{-3}$	11.9	$3.1 \cdot 10^{-6}$	19.2
	**area**	$3.6 \cdot 10^{-3}$	16.8	$1.8 \cdot 10^{-6}$	12.4

Comparison of cell discrimination factors on-chip (AOC_{FSC} 0° and AOC_{SSC} 50°) and on a benchmark flow cytometer.

Table S3. **Cell discrimination factors.**

Monocyte vs. lymphocyte discrimination factor for the P2P amplitude and area population medians as measured on the benchmark and on-chip (AOC_{FSC} of 0° and AOC_{SSC} of 50°) flow cytometer.

	**Discrimination factor**

Device	Signal	P2P	Area
Benchmark flow cytometer	FSC	1.4	1.5
	SSC	2.5	2.7
On-chip flow cytometer	FSC	1.4	1.4
	SSC	1.5	1.4

Scatter plots for other collection grating configurations.
Various devices on-chip were designed to enable the collection of forward scattering in two modes: (1) axial light loss (ALL) at an AOC of 0° and (2) scattering mode at an AOC of 5°. In the case of ALL, the gratings were optimized for maximum coupling of the illumination light. In the scattering mode, the main illumination beam is passing next to the collection gratings, and the gratings were optimized for capturing only the scattered light at a small AOC. However, due to side lobes present in the illumination beam, some background light is still collected by the FSC gratings. SSC configurations were fabricated for collection at 40°, 50°, and 70°.

Figure S2 shows the histograms and scatter plot for 3 µm, 6 µm and 10 µm polystyrene beads measured on-chip with an AOC_{FSC} of 5° and an AOC_{SSC} of 70°. Here both the FSC and SSC are collected at an angle, as opposed to FSC in ALL mode. As a consequence, both FSC and SSC are affected by the distribution of the beads along the entire height of the microfluidic channel causing an overlap in the histograms. As such, the individual populations cannot be discriminated on the scatter plot of the bead mixture in panel **c**.

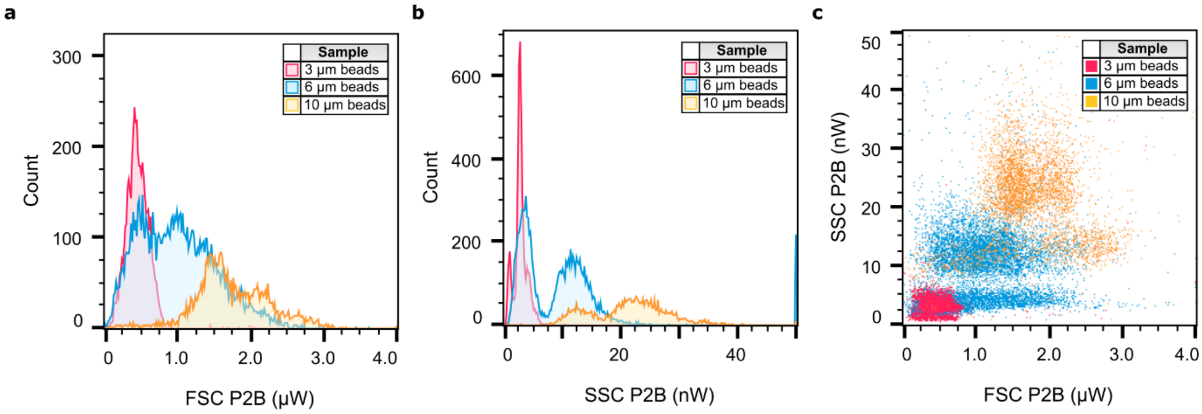

Figure S2. ***Counting and discrimination of polystyrene beads on-chip with an AOC_{FSC} of 5° and AOC_{SSC} of 70°.***
*Overlay of the peak-to-baseline (P2B) amplitude histograms corresponding to **a)** FSC and **b)** SSC of 3 µm, 6 µm, and 10 µm beads detected on-chip (AOC_{FSC} of 5° and AOC_{SSC} of 70°) and **c)** the corresponding SSC versus FSC overlay scatter plot.*

An example of a measurement of isolated cell populations and plain PBMCs on-chip with an interrogation point configuration of AOC_{FSC} of 5° and an AOC_{SSC} of 70° as well as benchmarking of the input sample on a benchmark flow cytometer is shown in Figure S3. For FSC recorded at an AOC of 5°, the SNR is higher (on average 150) than for FSC ALL (on average 65) due to a much-reduced background and noise. Yet, the cell population is smeared

out due to the sensitivity to a cell's z-position in the microfluidic channel. Discrimination between the individual lymphocyte and monocyte populations is by consequence less clear.

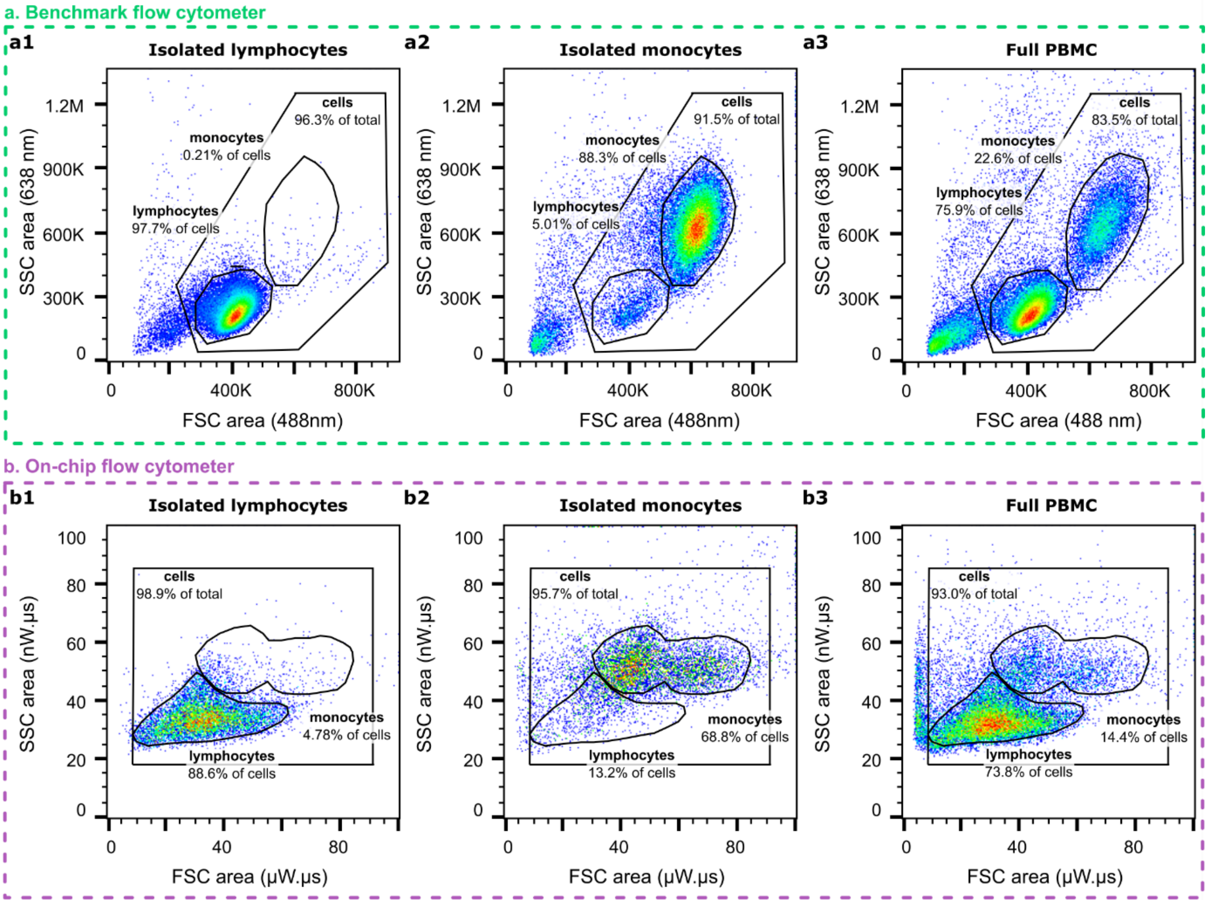

Figure S3. ***Scatter plots of the (isolated) PBMC populations on-chip with an AOC$_{FSC}$ of 5° and AOC$_{SSC}$ of 70° and benchmark with a benchmark flow cytometer.***

__a)__ Benchmark flow cytometer scatter plots of __(a1)__ isolated lymphocytes (54,692 events), __(a2)__ isolated monocytes (39,191 events), and __(a3)__ the complete PBMC sample before isolation (60,084 events). __b)__ On-chip flow cytometer (AOC$_{FSC}$ of 5° and AOC$_{SSC}$ of 70°) SSC area vs. FSC area and SSC area of __(b1)__ isolated lymphocytes (9,842 events), __(b2)__ isolated monocytes (8,505 events), and __(b3)__ the overlay of both isolated populations (21,100 events). For each scatter plot, the cell population is gated, as well as the monocyte and lymphocyte fraction therein.

The purities of the gated fractions on-chip are less than those measured for a device with an interrogation point configured with an AOC$_{FSC}$ of 0° and an AOC$_{SSC}$ of 50°. In Figure S3, 4.78% of the isolated lymphocytes overlap with the monocyte gate, and 13.2% of the monocyte sample ends up in the lymphocyte gate. Yet, for SSC, even though the collected intensity of the scattered light decreases with increasing AOC, the discrimination factor, on the other hand, increases. The highest discriminating factor, 1.6, was hence measured at an interrogation point with an AOC$_{SSC}$ of 70°. The trend of increasing discrimination factor is illustrated in Figure S4, which shows scatter plots of isolated lymphocyte and monocyte samples for three different configurations of the optical interrogation point, each with increasing AOC$_{SSC}$.

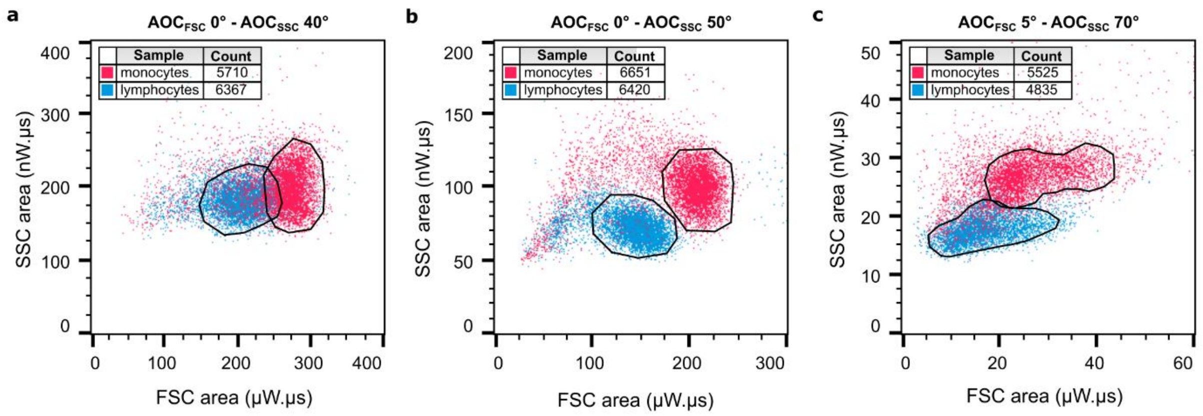

Figure S4. ***Effect of the collection grating configuration on the cell scatter plot.***

Overlay scatter plots of isolated lymphocyte and monocyte populations measured on-chip with an interrogation point configuration of ***a)*** *AOC_{FSC} 0° and AOC_{SSC} 40°,* ***b)*** *AOC_{FSC} 0° and AOC_{SSC} 50° and* ***c)*** *AOC_{FSC} 5° and AOC_{SSC} 70°. The gating was performed on the isolated populations.*

Fluorescence validation of cell classification by back-gating.

For validation of the accuracy of the cell classification, Table S4 summarizes the fractions of the fluorescent APC+ cell population when backgated onto the respective lymphocyte and monocyte scatter gates. The table shows the results for full PBMC with anti-CD3-APC labeled lymphocytes (also see Figure 7) as well as for full PBMC with anti-CD14-APC labeled monocytes. The accuracy of cell classification can be defined as the correctly predicted cell fraction, i.e. 89.24% for the PBMC with anti-CD3-APC labeled lymphocytes and 74.75% for the PBMC with anti-CD14-APC labeled monocytes. Yet, only 2.76% and 2.88% of the APC+ populations are mislabeled. The remaining 8% and 22.37%, respectively, are missed by the scatter gate but not wrongly labeled. In a subsequent cell sorting step, this fraction of missed cells reduces the recovery but not the sample purity, which is often key (e.g. in cell therapy manufacturing).

Table S4. ***Fluorescence validation of on-chip cell classification.***

Fractions of anti-CD3-APC labeled PBMC and anti-CD14-APC labeled PBMC as calculated for APC+ cells with a fluorescence intensity cut-off at 0.18 nW and 0.26 nW, respectively. as measured on the on-chip (AOC_{FSC} of 0° and AOCSSC of 50°) flow cytometer.

**Anti-CD3 Lymphocytes (APC+ vs. FSC area)**	**Fraction vs. cells (%)**	**Fraction vs. APC+ (%)**
APC+ fraction	47.4	100
APC+ fraction outside lymphocyte scatter gate	5.1	10.76
Correctly predicted fraction: APC+ fraction within the lymphocyte scatter gate	42.3	89.24
Impurity of APC+ fraction within the monocyte scatter gate	1.3	2.76
**Anti-CD14 Monocytes (APC+ vs. FSC area)**	**Fraction vs. cells (%)**	**Fraction vs. APC+ (%)**
APC+ fraction	19.8	100

APC+ fraction outside monocyte scatter gate	5.0	25.25
Correctly predicted fraction: APC+ fraction within the monocyte scatter gate	14.8	74.75
Impurity of APC+ fraction within the lymphocyte scatter gate	0.57	2.88

References

1. Ryan, C. G., Clayton, E., Griffin, W. L., Sie, S. H. & Cousens, D. R. SNIP, a statistics-sensitive background treatment for the quantitative analysis of PIXE spectra in geoscience applications. *Nuclear Inst. and Methods in Physics Research, B* **34**, (1988).
2. Butement, J. T. *et al.* Integrated optical waveguides and inertial focussing microfluidics in silica for microflow cytometry applications. *Journal of Micromechanics and Microengineering* **26**, (2016).
3. Zhang, J. *et al.* Fundamentals and applications of inertial microfluidics: a review. *Lab Chip* **16**, 10–34 (2016).
